# Towards the Prevention of Aminoglycoside-Related Hearing Loss

**DOI:** 10.3389/fncel.2017.00325

**Published:** 2017-10-18

**Authors:** Mary E. O’Sullivan, Adela Perez, Randy Lin, Autefeh Sajjadi, Anthony J. Ricci, Alan G. Cheng

**Affiliations:** ^1^Department of Otolaryngology-Head and Neck Surgery, Stanford University School of Medicine, Stanford, CA, United States; ^2^Department of Molecular and Cellular Physiology, Stanford University School of Medicine, Stanford, CA, United States

**Keywords:** aminoglycoside antibiotics, mechanotransducer channel, ribosome, ototoxicity, mRNA misreading

## Abstract

Aminoglycosides are potent antibiotics deployed worldwide despite their known side-effect of sensorineural hearing loss. The main etiology of this sensory deficit is death of inner ear sensory hair cells selectively triggered by aminoglycosides. For decades, research has sought to unravel the molecular events mediating sensory cell demise, emphasizing the roles of reactive oxygen species and their potentials as therapeutic targets. Studies in recent years have revealed candidate transport pathways including the mechanotransducer channel for drug entry into sensory cells. Once inside sensory cells, intracellular targets of aminoglycosides, such as the mitochondrial ribosomes, are beginning to be elucidated. Based on these results, less ototoxic aminoglycoside analogs are being generated and may serve as alternate antimicrobial agents. In this article, we review the latest findings on mechanisms of aminoglycoside entry into hair cells, their intracellular actions and potential therapeutic targets for preventing aminoglycoside ototoxicity.

## Introduction

Aminoglycosides are critical antimicrobials with potent activities against gram negative bacteria (World Health Organization, 2011). Over 10 million doses are consumed annually in the United States (Van Boeckel et al., 2014). In developed countries, aminoglycoside administration is tightly regulated and generally confined to inpatient settings where indications for their use include endocarditis, urinary tract infection, sepsis and other severe infections (Avent et al., 2011). In contrast, in some developing countries, usage is less restricted and substantially more commonplace. Moreover, because of their affordable cost and low incidence of antibiotic resistance, aminoglycosides are often selected in these countries as a first line of treatment (Van Boeckel et al., 2014).

Aminoglycosides are a large family of water-soluble, polycationic molecules that exist as either three- or four-ringed compounds. More specifically, aminoglycosides contain a common neamine core, composed of a six-member aminocyclitol ring (ring II) glycosidically linked to a glucosaminopyranose (ring I). Substitutions attached at either position 5 or 6 on ring II give rise to different aminoglycoside molecules that are categorized as either 4, 5 or 4, 6 aminoglycosides. To date, over 25 aminoglycoside compounds have been purified or synthesized (NCBI PubChem Compound Database, 2017). Aminoglycosides that are more commonly used clinically include neomycin, tobramycin, gentamicin and amikacin (Arya, 2007; NCBI PubChem Compound Database, 2017).

A significant side effect of aminoglycoside administration is kidney damage (nephrotoxicity) and irreversible sensorineural hearing loss (ototoxicity). Nephrotoxicity is largely reversible, whilst hearing loss is permanent. Hearing loss occurs in a dose-dependent manner and the antibacterial efficacy, nephrotoxicity, and ototoxicity of these drugs correlate with aminoglycoside blood concentration (Lacy et al., 1998; Mingeot-Leclercq and Tulkens, 1999; Chen et al., 2013). Hence, aminoglycosides are typically administered in inpatient settings and therapeutic drug monitoring of aminoglycoside levels in the blood is routinely performed in most developed countries (Freeman et al., 1997; Smyth et al., 2005; Agency for Healthcare Research and Quality, 2012). Because aminoglycosides are not metabolized and excreted exclusively through the kidneys, drug monitoring is particularly important among patients with compromised kidney function.

Even when aminoglycoside blood concentrations are within the recommended therapeutic range, ototoxicity can still occur. Some studies have indicated that cumulative duration of therapy, rather than peak and trough levels of drugs, is predictive of ototoxicity (Beaubien et al., 1989; Cheng et al., 2009; Modongo et al., 2015). Moreover, in a subset of patients with the mitochondrial DNA mutation m.1555A>G, hearing loss can occur following a single dose (Usami et al., 1998). Individuals with this mutation may otherwise have hearing that is within normal limits unless they have been exposed to aminoglycosides (Bitner-Glindzicz et al., 2009; Rahman et al., 2012). These studies illustrate how patients’ genotypes can influence the penetrance of aminoglycoside-related hearing loss. To date, four mutations in the mitochondrial genome and four genes in the nuclear genome are reported to be involved in aminoglycoside-related hearing loss, these will be returned to later in this review article (Li and Guan, 2002; Bykhovskaya et al., 2004a,b; Guan et al., 2006; Guan, 2011).

Estimates of the prevalence of ototoxicity in patients vary widely across the literature, ranging between 2%–25% for hearing deficits and 1%–10% for vestibular dysfunction (Ariano et al., 2008; Huth et al., 2015). For patients who require multiple courses of intravenous aminoglycoside antibiotics (e.g., treatment of tuberculosis and cystic fibrosis patients) estimates are higher and may exceed 50% (Duggal and Sarkar, 2007; Waters et al., 2015). This variability is likely due to a variety of factors such as the sensitivity of the audiometric tests, the patient population studied, their comorbid conditions, previous aminoglycoside treatment, the specific aminoglycoside used, its dosage and duration of treatment (Al-Malky et al., 2011, 2015; Seddon et al., 2012; Zimmerman and Lahav, 2013).

After aminoglycoside exposure, the main cochlear pathology underlying drug-induced hearing loss is sensory hair cell loss. Sensory hair cells are mechanoreceptors required for hearing and balance functions. In the cochlea, they are tonotopically arranged such that high frequency sounds stimulate hair cells in the basal region and low frequency in the apical region. Early on in the disease process when hearing loss typically begins in the high frequency, hair cell loss is found in the basal region (Fausti et al., 1992). However, hearing loss can progress into the mid- and low frequency ranges with corresponding hair cell loss in those regions in the cochlea. As such, research efforts have focused on defining the mechanisms of aminoglycoside trafficking into hair cells and the intracellular events leading to their demise.

In the mammalian cochlea, the apical surface of sensory hair cells are located in an extracellular fluid compartment filled with a unique endolymph solution, comprised of high potassium (K^+^, replacing NaCl in normal extracellular fluid) and low calcium (Ca^2+^, 20 μM as compared to 1.2 mM in normal extracellular fluid; Sterkers et al., 1982, 1984). Also unique to this compartment is a high endocochlear potential (+80 mV), which serves to drive positively charged ions into the hair cell. The endolymph compartment is bordered on the lateral wall of the cochlea by the stria vascularis, which is essential for endolymph maintenance and production (Sterkers et al., 1982; Salt and Plontke, 2005). The endocochlear potential, established by high concentration of K^+^ ions (160 mM), is the major driver for sensory transduction. During sound stimulation, K^+^ ions flow down an electrochemical gradient into hair cells through mechanically-gated channels located in sensory hair cell bundles. These unique, mechanically sensitive channels are non-selective cation carriers, allowing other large positively charged molecules, like aminoglycosides, to enter the hair cell.

Aminoglycosides were first discovered and used clinically in the 1940s, yet today and over 50 years later, preventative options remain largely limited to monitoring of dose and treatment duration. Decades of research have established fundamental knowledge and we refer the avid readers to other excellent reviews (Huth et al., 2011; Stawicki et al., 2015; Wong and Ryan, 2015). This review article will highlight two main research areas aminoglycoside transport and intracellular targets, as well as detailing novel mechanisms, and their respective potentials and limitations as therapeutic targets.

### Targeting the Stria Vascularis to Prevent Ototoxicity

The stria vascularis houses the major blood-labyrinth barrier (BLB)–a highly specialized capillary network that controls exchanges between blood and the interstitial space in the cochlea. The BLB is one of the first sites of aminoglycoside entry from the blood into the inner ear, a concept first proposed by Hawkins (1973) (Dai and Steyger, 2008; Wang et al., 2010). The BLB is strongly influenced by physiological factors such as active and passive membrane functions, ion channels, blood flow, inflammation, free radicals and possibly noise exposure (Abbott and Blakley, 2007; Shi, 2016). This structure is capable of affecting the pharmacokinetics of aminoglycosides in the inner ear, supported by evidence that endotoxin-mediated inflammation enhances aminoglycoside trafficking across the BLB and potentiates cochlear uptake of aminoglycosides and permanent hearing loss in mice (Koo et al., 2015). Data also shows that melatonin exacerbates aminoglycoside ototoxicity *in vivo*, among its many roles in the body melatonin acts as a vasoconstrictor and vasodilator (Erdem et al., 2005).

Aminoglycosides conjugated to fluorescent labels have been developed and proven to be a powerful tool in the study of aminoglycoside trafficking from the blood into the endolymph. The most commonly used fluorescent aminoglycoside is gentamicin sulfate conjugated to the Texas red fluorophore, so called Gentamicin Texas Red (GTTR; Sandoval et al., 1998; Dai and Steyger, 2008). Notably, owing the variable chemical composition of compounds marketed as “Gentamicin Sulfate”, GTTR is not one pure compound but contains a mixture of gentamicin compounds differing by methyl, amine and hydroxyl groups (Antec, 2014). Chemical limitations of the current synthesis method mean that the site to which the Texas red fluorophore is attached is unknown, moreover, it could be conjugated to one or more of the six amines on the structure of gentamicin (Sandoval et al., 1998; Dai and Steyger, 2008). Whilst the compound itself has purity and fluorophore-related limitations, the GTTR compound is an incredibly useful tool in tracing aminoglycoside transport in the cochlea.

Studies show that GTTR systemically administered is first found in strial capillaries before being distributed to the marginal cells in the stria vascularis and the endolymph (Dai and Steyger, 2008; Wang and Steyger, 2009). As noted earlier, aminoglycoside treatment is associated with nephrotoxicity and ototoxicity. Because of the commonality in genes expressed between the two tissues, it is hypothesized that agents that trigger a pathological change in one may similarly affect the other. In particular, there are similarities between the stria vascularis in the cochlea and the proximal tubules and loop of Henle of the kidney (Quick et al., 1973; Jentsch et al., 2004; Fahlke and Fischer, 2010). For example, both the stria vascularis and kidneys contain high levels of megalin, a vitamin D transporter that may play a role in aminoglycoside transport into the endolymph and megalin deficiency confers protection from aminoglycoside-induced nephrotoxicity (Mizuta et al., 1999; Schmitz et al., 2002). Closure or manipulation of vitamin D transporters or the stria vascularis as an entry pathway is not yet investigated but could serve as a mechanism to prevent aminoglycoside ototoxicity.

### Targeting Mechanotransducer Channel Mediated Entry to Prevent Ototoxicity

As an otoprotective strategy, preventing aminoglycoside entry into hair cells has emerged as another research focus in recent years (Huth et al., 2015). Breakthroughs following this strategy stem from the knowledge pool gained on the mechanotransduction (MET) channel, an area we will briefly review below.

Aminoglycosides enter hair cells via the MET channel, a non-specific cation channel at the tips of the hair cell stereocilia that opens and closes in response to hair bundle deflection (Alharazneh et al., 2011; Vu et al., 2013; Marcotti et al., 2016). The molecular structure of this mechanically-gated channel has been the subject of much research and the protein composition of the MET channel in hair cells remains unclear (Fettiplace and Kim, 2014; Zhao and Müller, 2015). Nonetheless, studies have extensively characterized the biophysical properties of the MET channel and estimated its pore opening to be <1.70 nm and its narrowest portion at 1.25 nm (Farris et al., 2004; Marcotti et al., 2005). The MET channel pore dimension is thus large enough to accommodate the passage of dihydrostreptomycin, a four-ringed aminoglycoside whose end-on diameter was estimated at 0.8 nm (Marcotti et al., 2005). Whilst dihydrostreptomycin can fit through this pore, it also acts as a permeant channel blocker, i.e., it forms a temporary block that is reduced by high extracellular calcium, and is voltage dependent, decreasing at extreme positive and negative potentials (Gale et al., 2001; Ricci, 2002; Ricci et al., 2002; Marcotti et al., 2005). The ability of different aminoglycosides to serve as permeant blockers of the MET channel likely varies as a function of the chemical size and charge. An electrophysiology study showing a side-by-side comparison of aminoglycosides in terms of size and charge is lacking, only sisomicin and a novel derivative have been studied independently (Farris et al., 2004; Huth et al., 2015). Whether channel permeation by aminoglycosides correlates with the level of ototoxicity remains to be explored.

Aminoglycosides more readily enter hair cells in the base than the apex corresponding to larger transduction currents at the cochlear base, and the larger single channel conductance in basal outer hair cells (Ricci et al., 2003; Waguespack and Ricci, 2005; Beurg et al., 2006). This is also consistent with the findings that basal hair cells are more susceptible to damage by aminoglycosides and that patients experience hearing loss beginning at the high frequencies.

**Figure 1:**
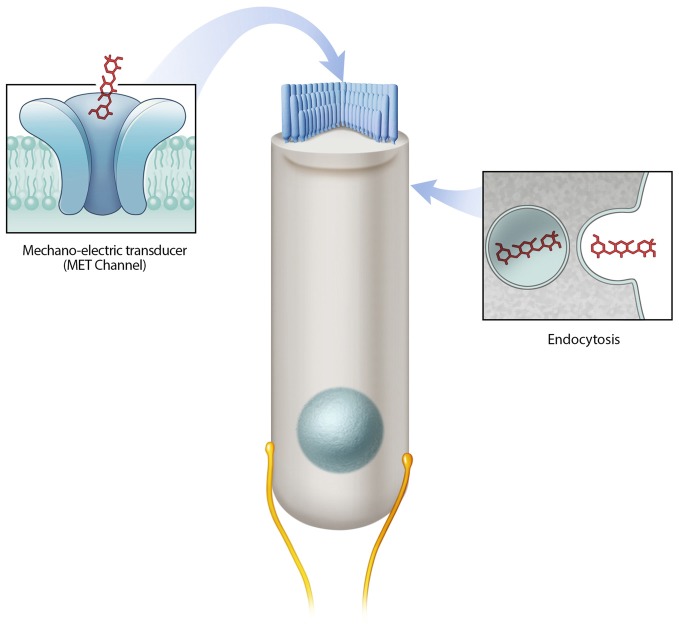
Mechanisms of aminoglycoside hair cell entry. Two prevailing mechanisms are reported to allow aminoglycosides to enter hair cells—the mechano-electric transducer (MET) channel and endocytosis. The MET channel located at the tips of hair cell stereocilia mediates the flow of ions (including aminoglycosides) from the endolymph compartment into hair cells. Endocytosis mediated drug entry occurs via the invagination of the cell membrane.

A number of critical experiments demonstrate the importance of the MET channel as a mechanism of aminoglycoside hair cell entry. First, using the GTTR compound, Alharazneh et al. (2011) found that aminoglycosides are rapidly taken up into hair cells within minutes in rat cochlear explants. Moreover a variety of MET channel blockers were observed to protect against hair cell loss *in vitro*, leading to the suggestion that channel blockers can be otoprotective. Blocking MET channels (e.g., with amiloride, quinine or curare) prevents aminoglycoside entry into hair cells and confers hair cell protection from various aminoglycosides (Alharazneh et al., 2011). However, the potential issue with using channel blockers for otoprotection is that many of the blockers used are toxic to humans and thus are not suitable as therapeutic agents. Second, the data indicating that calcium competes with aminoglycosides as a permeant blocker of the MET channel supports the role of MET channels in aminoglycoside entry (Ricci, 2002; Coffin et al., 2009). In mammalian studies, low calcium levels result in more robust drug uptake and toxicity and conversely, higher calcium levels, which decrease MET channel currents, were otoprotective (Ricci, 2002; Ricci et al., 2002; Vu et al., 2013). In other studies, decreased GTTR uptake is observed using high calcium in the extracellular solution, which also leads to a reduction in hair cell loss caused by aminoglycoside treatment in both the lateral line *in vivo* and cochlea *in vitro* (Coffin et al., 2009; Wang and Steyger, 2009; Ou et al., 2012). Thirdly, several genetic models with reduced or abolished MET channel function also demonstrate reduced aminoglycoside uptake and/or toxicity, such as those that result from Cadherin23 deletion, Myosin7a deletion, and Transmembrane channel-like proteins 1 and 2 double knockouts (Wang and Steyger, 2009; Kawashima et al., 2011; Vu et al., 2013; Marcotti et al., 2016). Taken together, these studies indicate that the MET channel is a major entry route for aminoglycosides into hair cells and that this mode of entry is required for hair cell toxicity (Figure 1).

Leveraging knowledge of the MET channel biophysical properties, Huth et al. (2015) designed a new version of modified aminoglycosides and found that sisomicin derivatives were less toxic to hair cells *in vitro* and *in vivo* than the parent aminoglycoside. When compared to sisomicin, the lead compound N1MS was found to permeate the MET channel to a lesser degree than the parent compound sisomicin by electrophysiological measurements (Kd = 65.0 ± 17.7 vs. 96.1 ± 9.9 μM, respectively; Huth et al., 2015). However, they found that altering aminoglycosides could decrease their antimicrobial activities, or in the case of N1MS, shift its spectrum of activities (Huth et al., 2015). While this study shows that it is possible to separate aminoglycoside entry into hair cells from entry into bacteria, more work is needed to gain insights into the relationship between aminoglycoside structure and antimicrobial actions, in particular bacterial uptake. While different mechanisms of aminoglycoside uptake likely exist between bacteria and sensory hair cells, it is possible intracellular actions (e.g., aminoglycoside-ribosome interactions) between the two cell types are similar. We will discuss aminoglycoside-ribosome binding later in this review article. In general, these recent studies provide proof-of-principal data that ototoxicity and antimicrobial activity can be separated and also that by preventing aminoglycoside entry via MET channels, ototoxicity is greatly reduced.

### Targeting Alternative Entry Routes to Prevent Ototoxicity

Endocytosis is another route of entry for aminoglycosides into hair cells (Figure 1), however, relative to MET channel-mediated uptake it is slower (Hashino and Shero, 1995; Alharazneh et al., 2011). Immunogold-labeling and GTTR-based experiments show the presence of aminoglycosides in membrane-bound vesicles beneath the cell surface of hair cells (Hashino and Shero, 1995). The number of aminoglycoside-containing vesicles appears to increase over time with a subset increasing in size, presumably as a result of aggregation (Hashino and Shero, 1995). Recent live imaging studies reveal that both endocytic and non-endocytic uptake of aminoglycosides occur, and that both mechanisms can result in accumulation of drug in lysosomes (Nagai and Takano, 2014; Hailey et al., 2017). Lysosomes are the primary degradative compartment of eukaryotic cells (Giraldo et al., 2014; Perera and Zoncu, 2016). Notably, identification of compounds in vesicles does not causally link to the mechanism of uptake as entry via MET channels or endocytosis. Evidence supporting the role of endocytosis in aminoglycoside ototoxicity include observations made in the kidney where aminoglycosides are endocytosed and subsequently accumulate within lysosomes of renal proximal tubular cells, leading to cell death (Moestrup et al., 1995; Nagai and Takano, 2014).

In addition to the MET channels, other ion channels are implicated in mediating aminoglycoside entry into hair cells. One such family is the transient receptor potential (TRP) channels, which are large calcium-permeant, cationic channels (Myrdal and Steyger, 2005; Karasawa et al., 2008). While various channels are implicated in mediating aminoglycoside transport in renal tubular cells *in vitro*, their exact roles in mediating aminoglycoside ototoxicity are unclear (Myrdal and Steyger, 2005; Karasawa et al., 2008). For example, activated TRPA1 channels allow GTTR entry when the MET channel is blocked (Stepanyan et al., 2011). Since TRPA1 channels can be activated by lipid peroxidation products, it is proposed that it can amplify aminoglycoside uptake in addition to MET channel in damaged hair cells (Stepanyan et al., 2011). Another candidate modulator is the chloride/bicarbonate exchanger Slc4a1b, which mediates aminoglycoside uptake in the kidney (Stehberger et al., 2007; Alper, 2009) and in hair cells in the zebrafish lateral line (Hailey et al., 2017). Although the Slc family proteins are expressed in the stria vascularis in the mammalian cochlea, their exact roles in aminoglycoside transport are unknown and warrant further investigation (Stankovic et al., 1997).

As a strategy for preventing ototoxicity, targeting endocytosis and other ion channels is challenging as these pathways are not specific to hair cells, nor do these entry mechanisms appear to be the most predominant in the hair cell. Moreover, preventing endocytosis pharmacologically may have off-target effects. As we move further down the chain of events in aminoglycoside ototoxicity the issue of off-target effects becomes larger and the challenge of preventing damage becomes arguably greater.

### Targeting Intracellular Mechanisms to Prevent Ototoxicity

A plethora of cellular structures and metabolic pathways are implicated in aminoglycoside ototoxicity. Building on a large body of literature on the roles of reactive oxygen species, recent studies aiming at unraveling these mechanisms at the organelle (mitochondria and endoplasmic reticulum (ER)) and other subcellular levels (ribosome, membrane lipids and potassium channel interactions) may open the door to preventing ototoxicity (Huth et al., 2015; Stawicki et al., 2015; Wong and Ryan, 2015). As it is difficult to distinguish between mechanisms that are primary and secondary in hair cell stress, we have grouped the mechanisms by target.

**Figure 2:**
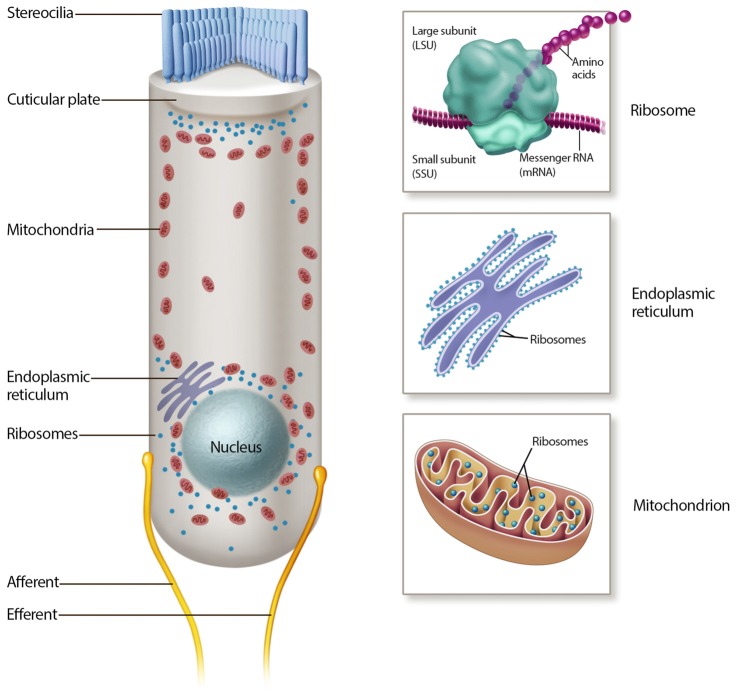
Mechanisms of aminoglycoside intracellular action. Many intracellular mechanisms may be involved in cochlear hair cell death, including mitochondrial and cytosolic ribotoxicity, mitochondrial and endoplasmic reticulum (ER) calcium signaling, lipid interactions and reactive oxygen species (ROS) production.

Once inside the hair cell, aminoglycosides rapidly fill the cytoplasm (Alharazneh et al., 2011). As cationic molecules, aminoglycosides are drawn to negatively charged molecules such as DNA, RNA, negatively charged phospholipids and cationic binding sites (basic residues) in proteins. In addition to building up in the cytoplasm, reports of ER and mitochondrial stress suggest that aminoglycosides accumulate within these organelles (Figure 2; Guan et al., 2000; Esterberg et al., 2014). These organelles are concentrated at two key functional zones in the hair cell, the apical region below the cuticular plate and hair bundle, and the basolateral region near the synaptic complex.

The mitochondrion is a dynamic, double membrane bound organelle that contains its own genome and translational apparatus (mitochondrial ribosome or mitoribosome; Anderson et al., 1981). The mtDNA is a closed circular molecule of 16,569 nucleotides that encodes proteins involved in oxidative phosphorylation (OXPHOS; Anderson et al., 1981). This organelle has many functions, most notably the production of ATP via OXPHOS, a pathway responsible for producing over 90% of cellular ATP. Other energetic mitochondrial functions are reviewed elsewhere (Wallace et al., 2010). On the other hand, the ER is one of the largest organelles in the eukaryotic cells (Oakes and Papa, 2015). It is a network of branching tubules and flattened sacs interconnected through enclosed spaces called the ER lumen, which plays major roles in protein folding and calcium regulation (Oakes and Papa, 2015).

### Targeting the Ribosome to Prevent Ototoxicity

As in bacteria, an intracellular target for aminoglycosides in the mammalian cochlear hair cell is the ribosome. Ribosomes (~30% of eukaryotic cellular mass) decode genetic information and convert it into proteins (Kramer et al., 2009). Each cell contains up to 10^6^ ribosomes and 5–9 amino acids are incorporated into proteins per second (Kramer et al., 2009). In bacteria, aminoglycosides bind to the small ribosomal subunit, where they disrupt the rate and accuracy of protein synthesis (Ogle and Ramakrishnan, 2005). In humans, a longstanding hypothesis of aminoglycoside ototoxicity is that hair cell dysfunction arises from a similar mechanism of action due to structural resemblances between eukaryotic and bacterial ribosomes (Hutchin et al., 1993; Prezant et al., 1993; Guan et al., 2000; Hobbie et al., 2006, 2008a,b; Greber et al., 2015).

**Figure 3:**
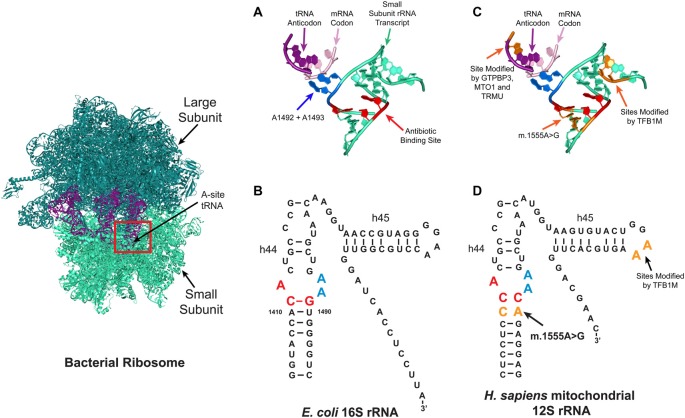
Aminoglycoside ribosome binding. Aminoglycosides can bind to several sites on ribosomes, with the primary prokaryotic binding site reported to be a conserved pocket in the A-site (red box) formed by helix h44 of the 16S rRNA in the small ribosomal subunit. At this site, the correct tRNA anti-codon is matched with the correct mRNA codon. **(A,B)** Tertiary **(A)** and secondary **(B)** structures of the bacterial A-site. In bacteria, aminoglycosides bind to three conserved nucleotides A1408, C1409 and G1491 (red). **(C,D)** Schematic depicting the tertiary **(C)** and secondary **(D)** structures of the mitochondrial A-site. In the human mitochondrial ribosome, patients with the m.1555A>G mutation have a new base pair formed which makes it resemble the bacterial ribosome. Four nuclear genes are reported to modify the penetrance of aminoglycoside related hearing loss (TRMU, MTO1, GTPBP3 and TFB1M), the tRNA and rRNA nucleotides modified by these enzymes are shown in orange.

Of the two types of ribosome (mitochondrial and cytoplasmic) in the eukaryotic cell, the mitoribosome was implicated in aminoglycoside ototoxicity in 1993 (Hutchin et al., 1993; Prezant et al., 1993). The strongest evidence supporting the role of the mitoribosome in aminoglycoside ototoxicity is antibiotic hypersensitivity in patients with the m.1555A>G mitochondrial DNA mutation (Hutchin et al., 1993; Prezant et al., 1993; Estivill et al., 1998). Patients with this mutation located adjacent to the aminoglycoside ribosomal binding site are particularly susceptible to aminoglycoside induced hearing loss. While the mitochondrial ribosomal transcript in eukaryotic cells normally differs from that in the bacteria, the m.1555A>G mutation re-establishes one of the evolutionarily lost bacterial-like base pairs in the mitoribosome (Figure 3). Consequently, the structure of mutant mitochondrial ribosomal transcript resembles that of the *E. coli* transcript, rendering the mitochondrial decoding site more susceptible to aminoglycoside binding, resulting in translational impairment and possibly increased sensitivity to aminoglycoside toxicity (Hutchin et al., 1993; Böttger, 2010). In patients with the m.1555A>G mutation, hearing loss can occur following a single dose (Usami et al., 1998).

Functional studies also support the role of the mitoribosome in aminoglycoside ototoxicity. In human blood and skin-derived cell lines aminoglycoside treatment impairs mitochondrial protein synthesis (Guan et al., 2000; Giordano et al., 2002). Also a series of cell free assays using ribosomal constructs show that aminoglycosides have higher binding affinities for m.1555G ribosomes, which are less accurate at selecting the cognate tRNA when exposed to aminoglycosides (Hobbie et al., 2008b). Based on these results, it is hypothesized that cochlear hair cell death occurs when the level of translation decreases to below a certain threshold level (Guan et al., 2000; Giordano et al., 2002).

Furthermore, genetic studies lend additional support for mitoribosomal involvement by indicating that the variable penetrance of aminoglycoside-related hearing loss is due to the nuclear genetic background. Currently, four nuclear-encoded modifiers have been identified: mitochondrial transcription optimization 1 (MTO1); GTP binding protein 3 (GTPBP3); 5-methylaminomethyl-2-thiouridylate methyltransferase (TRMU); and mitochondrial transcription factor 1 (TFB1M; Li and Guan, 2002; Bykhovskaya et al., 2004a,b; Guan et al., 2006). GTPBP3, MTO1 and TRMU are hypothesized to alter the penetrance of aminoglycoside-related hearing loss by altering the accuracy of tRNA anti-codon mRNA codon interactions in the A-site (Figure 3; Li and Guan, 2002; Bykhovskaya et al., 2004b; Guan et al., 2006). The TFB1M enzyme modifies two, highly conserved, adjacent residues on the 3’ end of the 16S rRNA (Figure 3), but it remains unclear how TFB1M modifies aminoglycoside-related hearing loss (Seidel-Rogol et al., 2003; Cotney et al., 2009; Metodiev et al., 2009; Raimundo et al., 2012; Sharoyko et al., 2014; Lee et al., 2015; O’Sullivan et al., 2015).

While the established model of aminoglycoside ototoxicity suggests that mitochondrial ribotoxicity triggers hearing loss, there is a growing body of evidence suggesting that the cytosolic ribosome may also be affected by aminoglycoside (Nudelman et al., 2010; Kandasamy et al., 2012; Francis et al., 2013; Shulman et al., 2014). The cytosolic ribosome is responsible for the synthesis of over 20,000 cytosolic proteins and its composition, origin and function are different from its mitochondrial and bacterial counterparts (Ramakrishnan, 2002; Greber et al., 2015; Khatter et al., 2015). Francis et al. (2013) showed that the degree of aminoglycoside toxicity correlates closely with the extent of inhibition of cytoplasmic protein synthesis using an *in vitro* model. A prime example of aminoglycoside cyto-ribosome interaction is the clinical application of aminoglycosides in “codon-read through” therapy as a treatment for genetic diseases such as Rett syndrome, cystic fibrosis and Duchenne muscular dystrophy (Burke and Mogg, 1985; Nudelman et al., 2010; Baradaran-Heravi et al., 2017). Here, the concept is that synthesis errors can suppress otherwise deleterious mutations, such as premature stop-codon mutations.

Current ribotoxicity paradigms indicate that aminoglycosides kill bacteria by binding to the bacterial ribosome causing mistranslation. It is reduced aminoglycoside affinities to the corresponding eukaryotic ribosome as well as differences in cell entry that provide the rationale for bacterial specificity of aminoglycosides. Several studies have characterized and developed aminoglycosides with lower affinities for the mitochondrial ribosome (Matt et al., 2012; Perez-Fernandez et al., 2014). For example, the veterinary aminoglycoside, apramycin, is less ototoxic to hair cells *in vitro* and *in vivo*, and the lower ototoxicity profile of this aminoglycoside can be attributed to the lower affinity of this drug for the binding pocket in the mitochondrial ribosome (Matt et al., 2012). To further the development of less ototoxic aminoglycosides, more detailed studies are needed to evaluate the contribution of mitochondrial and cytosolic translational inhibition in aminoglycoside ototoxicity as it is unclear which mechanism predominates *in vivo*.

In addition to modifying the aminoglycoside backbone to prevent ototoxicity, two other strategies may be employed to prevent the effects of aminoglycoside-induced ribotoxicity: increased ribosomal accuracy and increased tolerance by the cell for ribosomal errors. In the current model of ribosome related ototoxicity an increase in errors as a result of aminoglycosides triggers cellular dysfunction. Amino-acid mis-incorporations during translation naturally occur, one in every 1000–10,000 codons translated is mistranslated (Drummond and Wilke, 2009). Studies indicate that both bacterial and mammalian ribosomes have the potential to switch to hyper-accurate states (Ruusala et al., 1984; Lodmell and Dahlberg, 1997). Moreover, in cell lines carrying mitochondrial translational defects, amino acid supplementation is reported to increase the fidelity of mitochondrial translation and can rescue translation defects *in vitro* (Boczonadi et al., 2013).

### Targeting the Other Intracellular Events to Prevent Ototoxicity

In addition to interaction with the mitochondrial and cytosolic ribosomes, many potentially overlapping cellular events likely occur upon aminoglycoside entry into the hair cell. We will briefly review these areas in this section.

#### Calcium Signaling

Calcium signaling is a key component in aminoglycoside induced cell death (Esterberg et al., 2013, 2014; Hailey et al., 2017). Calcium ions are important signaling molecules and in hair cells, precise regulation of Ca^2+^ concentrations is critical, e.g., Ca^2+^ defines the open probability of the MET channel (Ricci et al., 1998). Recent *in vivo* zebrafish experiments show Ca^2+^ levels are elevated in the ER following neomycin treatment and that the flow of Ca^2+^ between the ER and mitochondrion is a key event in aminoglycoside ototoxicity (Esterberg et al., 2013, 2014; Hailey et al., 2017). Esterberg et al. (2013) showed that in aminoglycoside-treated hair cells from the zebrafish lateral line, spikes in Ca^2+^ levels in the ER are followed by similar spikes in the mitochondria. Ca^2+^ spikes increase mitochondrial respiration, reactive oxygen species production and lead to a collapse in mitochondrial membrane potential, release of Ca^2+^ from these intracellular stores and subsequent cell death (Esterberg et al., 2013, 2014; Hailey et al., 2017).

#### Lysosomal Degradation

The lysosome controls the degradation and recycling of proteins and polysaccharides by intracellular autophagy (Giraldo et al., 2014; Perera and Zoncu, 2016). Damaged material is engulfed by autophagosomes via endocytic pathways, which then fuse with the lysosome and result in the recycling of amino acids and monosaccharides (Giraldo et al., 2014; Perera and Zoncu, 2016). Evidence for lysosomal involvement in ototoxicity arose from mechanisms associated with nephrotoxicity where aminoglycosides are known to be transported and accumulate within lysosomes of renal proximal tubular cells causing injury and necrosis (Moestrup et al., 1995). In aminoglycoside-treated hair cells, the antibiotic is also detectable in lysosomes (Hashino and Shero, 1995; Hashino et al., 1997, 2000; Steyger et al., 2003; Dai et al., 2006; Hailey et al., 2017). Interestingly, pharmacological inhibition of aminoglycoside uptake into lysosomes exacerbated hair cell death, implicating an important role of lysosomal degradation (Hailey et al., 2017).

#### Interactions with the Plasma Membrane, PIP2s and Potassium Channels

Aminoglycosides can also directly interact with membrane lipids. For example, they can induce rapid changes to the hair cell plasma membrane including phosphotidylserine externalization and membrane blebbing on the apical surface (Richardson and Russell, 1991; Goodyear et al., 2008). Aminoglycosides also bind to and alter the levels of phosphoinositides phosphatidylinositol-4,5-bisphosphate (PIP2) and phosphatidylinositol l-3,4,5-trisphosphate (PIP3; Schacht, 1976; Gabev et al., 1989). As PIP2 controls transduction and adaptation by hair cells, this may in turn affect drug uptake and hair cell viability (Hirono et al., 2004). Biochemical studies demonstrate that several aminoglycoside members bind to negatively charged phospholipid bilayers and inhibit the activity of lysosomal enzymes (Brasseur et al., 1984, 1985). In bacteria, studies show that aminoglycosides also bind to lipid bilayers (Sautrey et al., 2016).

In the mammalian cochlea, the potassium channel KCNQ4 is highly expressed in outer hair cells and KCNQ4 mutations cause human hereditary hearing loss (Kubisch et al., 1999; Kharkovets et al., 2000, 2006). One proposed etiology of hearing loss is outer hair cell death caused by KCNQ4 deficiency (Kharkovets et al., 2000, 2006; Nouvian et al., 2003). As the activity of several KCNQ isoforms including KCNQ4 is linked to PIP2 metabolism aminoglycosides may indirectly cause KCNQ4 dysfunction by disrupting PIP2 homeostasis and in turn induce outer hair cell death (Li et al., 2005; Suh et al., 2006). In support of this possible mechanism, like the KCNQ4 deficient cochlea, aminoglycoside ototoxicity typically displays outer hair cell loss in a basal-to-apical gradient (Kubisch et al., 1999; Kharkovets et al., 2000, 2006).

#### Free Radical Production

Changes in free radicals contribute to signaling and cell death pathways. Free radicals encompass both reactive oxygen species (ROS) and reactive nitrogen species (RNS), and it is the odd number of electron(s) of a free radical that makes it unstable, short-lived and highly reactive (Forge and Schacht, 2000; Huth et al., 2011; Phaniendra et al., 2015). Both ROS and RNS are required for normal cellular function (e.g., intracellular signaling) and their damaging capabilities are tightly regulated by the antioxidant system. However, when the antioxidant defense system is overwhelmed, free-radical induced oxidative stress occurs damaging the integrity of various cellular components including lipids, proteins and DNA, thus contributing to cell death. Aminoglycoside-induced ROS production has been heavily studied. ROS are electrophilic molecules generated by the partial reduction of oxygen to form superoxide, hydrogen peroxide, and hydroxyl radicals. There are multiple sources of ROS in the cell, including mitochondria, peroxisomes, the ER, and NADPH oxidase enzymes (Brand, 2010; Phaniendra et al., 2015). In most cell types, the mitochondrial OXPHOS system is the largest contributor to intracellular oxidant production, with superoxide radicals being produced at two major sites during electron transport, namely Complex I (NADH dehydrogenase) and Complex III (ubiquinone cytochrome c reductase; Brand, 2010; Jastroch et al., 2010).

Aside from drug monitoring, efforts aimed at preventing aminoglycoside ototoxicity have focused on mitigating the damage effects of ROS since *in vivo* and *in vitro* studies show ROS production in cochlear hair cells following aminoglycoside treatment (Rybak and Whitworth, 2005; Shulman et al., 2014; Esterberg et al., 2016). Protection against aminoglycoside ototoxicity has also been demonstrated by a wide array of antioxidants (e.g., lipoic acid, Coenzyme Q10, N-acetylcysteine, vitamin E and salicylates; Rybak and Whitworth, 2005; Sha et al., 2006; Noack et al., 2017). A growing number of reports indicate that the mechanism of ROS generation and the role of ROS in aminoglycoside induced cell death may be more complicated than initially proposed (Francis et al., 2013; Majumder et al., 2015; Esterberg et al., 2016). For example, *in vitro* experiments show the depletion of glutathione (a major ROS scavenger) does not increase susceptibility to aminoglycoside induced cell death suggesting alternate roles for ROS (Majumder et al., 2015).

## Discussion

Aminoglycoside ototoxicity represents one of the most common, preventable forms of drug-related hearing loss worldwide. Studies in recent years have catapulted our understanding of this disease entity and revealed novel therapeutic approaches. Many unanswered questions remain thus impeding antibiotic/otoprotectant design and development. To help coordinate a targeted approach to tackle these questions, we will discuss key focus areas that can hopefully direct efforts to accelerate the prevention of aminoglycoside ototoxicity.

1. *How are novel aminoglycosides less ototoxic?* It remains incompletely understood how novel aminoglycosides that have been developed are less ototoxic (Matt et al., 2012; Perez-Fernandez et al., 2014; Huth et al., 2015). Not only will this shape the development of the next generation of safer aminoglycoside antibiotics but it will also be relevant to those using aminoglycosides for codon-read-through therapies for rare diseases (Baradaran-Heravi et al., 2017). Specifically, one will need to establish whether compounds with lower affinities for the mitochondrial ribosome also show less entry into hair cells (Matt et al., 2012; Perez-Fernandez et al., 2014). Conversely, understanding whether compounds less readily entering hair cells are also toxic to the human ribosome is equally important (Huth et al., 2015). For this work, an *in vitro* screening assay using cochleae from an m.1555A>G mouse model would be a powerful tool to assess the mitochondrial ribotoxicity of less toxic aminoglycosides, since hair cells with these mutations would be the most sensitive to compounds toxic to the mitochondrial ribosome. At present, this type of mouse model remains elusive owing to the limitations of mitochondrial DNA transformation (Holt and Jacobs, 2014).
2. *How do aminoglycosides travel from the blood to endolymph?* Our insights into how various medications including ototoxins such as aminoglycosides and the anti-neoplastic agent cisplatin traverse the blood-labyrinth-barrier is quite limited. This is in part due to our incomplete knowledge of candidate transporters expressed in the stria vascularis. Efforts by several laboratories to investigate gene expression and function should aid our understanding of aminoglycoside entry into the endolymph, an area that holds significant promise in preventing aminoglycoside ototoxicity (Dai and Steyger, 2008; Li et al., 2011; Shi, 2016).
3. *How do aminoglycosides enter hair cells and bacteria?* Strategies focused on modifying aminoglycosides to prevent entry into hair cells hypothesize that differences in aminoglycoside uptake likely exist between bacteria and sensory hair cells. In hair cells both the MET channel and endocytosis are potential entry pathways (Figure 1). In the bacteria the membrane transporters porins and mechanosensitive channels are reported to allow aminoglycoside entry into gram negative cells (Silver, 2016). Should entry in both cell types occur predominantly through channel-mediated pathways, electrophysiological studies showing the permeation properties of different aminoglycosides ranked in terms of size, charge and hydrophobicity would be particularly valuable for novel drug design. Electrophysiology studies are somewhat limited by the size of bacteria (0.8–2 μm) but techniques have been developed to record the activities of mechanosensitive channels in several bacterial species, including the MscS and MscL channels (Moe et al., 1998; Blount et al., 1999; Martinac et al., 2013).
4. *What are primary and secondary intracellular interactions*? Many intracellular targets/mediators in hair cells are reported to be involved in aminoglycoside ototoxicity. This complexity has presented challenges in defining the molecular mechanisms, their temporal sequence, and also candidate therapeutic targets. It is also unclear if redundancy may exist and if aminoglycosides have multiple intracellular targets, making the efficacy of singular-target approaches uncertain. Nonetheless, one of the attractive targets for aminoglycosides is the ribosome, as biochemical and molecular evidence strongly suggest their interaction. Recent advances in cryo-electron microscopy may soon reveal aminoglycosides binding to the cytosolic and mitochondrial ribosomes *in situ*, results from which may inform binding patterns and potentially drug design/development (Greber et al., 2015).
5. *What is the true prevalence of ototoxicity?* As discussed previously, the reported incidence of aminoglycoside-induced hearing loss varies widely and establishing the true prevalence of ototoxicity following aminoglycoside exposure is key. Estimates of ototoxicity in patients range between 2%–25% for hearing deficits and 1%–10% for vestibular dysfunction (Ariano et al., 2008). To move the development of therapeutic options forward (e.g., otoprotective compounds or less ototoxic antibiotics) and help guide public interventions, such fundamental knowledge is critical. For example, close to 100% of patients with the m.1555A>G mutation are reported to develop hearing loss following aminoglycoside exposure. However, for other patients, the incidence is significantly lower. In order to formulate a national policy on genetic testing for the mitochondrial m.1555A>G mutation in the context of aminoglycoside usage in neonatal intensive care, the contribution of m.1555A>G to deafness in neonates who are likely to have received multiple courses of aminoglycoside antibiotics is currently being assessed (Bitner-Glindzicz et al., 2014).

## Conclusion

Tremendous genetic and functional work has shed light on the intricacies of aminoglycoside entry and action. This has facilitated the development of strategies with significant translational promise, in particular, the concept of modifying the aminoglycoside itself to prevent ototoxicity. The paradigm of modifying aminoglycoside antibiotics has been around for many years and nearly all antibiotics brought to market over the last 30 year have been variations on existing drugs (The Pew Charitable Trusts, 2016). However, it is only in recent years that this roadmap has been employed to reduce ototoxicity (Perez-Fernandez et al., 2014; Huth et al., 2015). In nature, aminoglycosides are modified by the living organisms (soil bacteria) that produce them. In industry, pharmaceutical companies have modified aminoglycosides (e.g., dibekacin, amikacin and netilmicin) synthetically to protect certain sites from aminoglycoside-modifying resistance enzymes and bolster antibacterial potency. Most recently, plazomicin, a semi-synthetic aminoglycoside with improved antibacterial efficacy has made it to a phase III clinical trials with readouts due in 2017 and 2018 (ClinicalTrials.gov, trial number NCT02486627 and NCT01970371). Should less ototoxic aminoglycosides with maintained or better antimicrobial potencies be developed, an accelerated track to clinical translation could be followed.

Going forward it is imperative that we continue to develop a better insight and scientific understanding of aminoglycoside entry and action, and that scientists modifying aminoglycosides to different ends continue to work together to develop compounds with better physiochemical properties. Whilst the development of life-saving antibiotics will always remain the priority, we owe it to our patients to continue to explore the possibility that a simple tag or chemical modification could limit the dramatic side effect that is aminoglycoside-related hearing loss—a global health problem.

## Author Contributions

MEO’S, AP, RL, AS, AGC and AJR: literature search; MEO’S, AGC and AJR: figure preparation and manuscript preparation.

## Conflict of Interest Statement

The authors declare that the research was conducted in the absence of any commercial or financial relationships that could be construed as a potential conflict of interest.
